# Preparation and Characterization of Casein–Soy Protein Hybrid Gels Cross-Linked by Transglutaminase

**DOI:** 10.3390/gels12030242

**Published:** 2026-03-13

**Authors:** Yan Ma, Juanjuan Chen, Meixia Yi, Xiaohui Xiong, Feng Xue, Chen Li

**Affiliations:** 1 College of Food Science and Light Industry, Nanjing Tech University, 30 Puzhu South Road, Nanjing 211816, China; 202361218225@njtech.edu.cn (Y.M.); 202461218274@njtech.edu.cn (J.C.); 18334993633@163.com (M.Y.); xxh@njtech.edu.cn (X.X.); 2 Jiangsu Key Laboratory of Medicinal Substance and Utilization of Fresh Chinese Medicine, Nanjing University of Chinese Medicine, Nanjing 210023, China; xuefeng@njucm.edu.cn

**Keywords:** casein–soy protein isolate, microstructure, gelation mechanism, physicochemical properties, intermolecular interactions

## Abstract

To enhance the gelling functionality of plant proteins, this study developed hybrid gels by blending casein with soy protein isolate (SPI) at various ratios using microbial transglutaminase (MTG) as a cross-linking catalyst. The gels were systematically characterized in terms of microstructure, water distribution, rheological and textural properties, secondary structure, and intermolecular interactions. Incorporation of casein significantly improved gel strength, water-holding capacity, and network uniformity. Notably, the 1:1 casein-to-SPI ratio yielded the highest performance, featuring extensive inter-protein cross-linking, an increased proportion of ordered secondary structures, and a finely porous matrix that effectively immobilized water. Mechanistically, MTG-catalyzed covalent bonding established the primary network scaffold, while hydrophobic interactions and disulfide bonds further stabilized the gel matrix. These findings demonstrate that MTG-induced Casein–SPI hybrid gels can enhance the functional properties of plant proteins and offer a viable strategy for designing sustainable protein-based food structures with tailored performance.

## 1. Introduction

The food system contributes approximately one-third of global anthropogenic greenhouse gas emissions, with animal-based production being a major driver [1]. In response, a global shift toward plant-based proteins is underway to promote more sustainable food systems [2]. However, the widespread adoption of plant proteins—such as soy protein isolate (SPI)—is often limited by their inherent functional shortcomings, including poor gelling properties, low water-holding capacity [3], and undesirable sensory attributes [4].

To address the above-mentioned limitations, blending plant proteins with animal-derived counterparts that exhibit superior gelling functionality presents a promising approach. Casein, the primary protein in milk, is a strong candidate for this role. Unlike many globular plant proteins, casein forms flexible, open-structured micelles with a high density of surface-exposed glutamine and lysine residues [5]. This unique conformation allows greater accessibility for enzymatic cross-linking and supports the formation of strong, elastic gel networks. In contrast, the compact, globular structure of SPI can shield its reactive sites, thereby limiting its gelation potential [6]. Thus, combining casein with SPI provides a compelling model system to explore how structural complementarity between an open-structured animal protein and a compact plant protein can enhance functional performance.

While the potential of protein hybrid gels is recognized, research has predominantly focused on blends involving whey protein [7], egg white protein [8], casein [9], or gelatin [10] with plant proteins. The specific interactions and gelation behavior of casein–SPI blends—particularly when enzyme-induced—remain underexplored. Conventional thermal gelation is often unsuitable for such systems, as proteins such as casein [11] and many plant proteins [12] exhibit high thermal stability. This necessitates intensive processing conditions that may compromise product quality. Microbial transglutaminase (MTG) offers a mild and efficient alternative. It is a highly effective food additive that improves the gelling properties of diverse food products due to its thermal stability, calcium-independent activation, and minimal deamidation activity [13]. By catalyzing isopeptide bond formation between glutamine and lysine residues, MTG can induce cold-set gelation, enhance network strength, and improve texture without impacting heat-sensitive components [14]. To date, MTG has been shown to improve the gel characteristics of surimi and surimi-based seafood [15] and modify the textural and rheological properties of dairy [16], meat [17], and bakery products [18]. Beyond its techno-functional benefits, MTG may offer health advantages by lowering the immunoreactivity of plant proteins, which could be exploited in dietary interventions for conditions like celiac disease [19]. Although MTG has been used to prepare hybrid protein systems, such as lentil protein–casein [20] and hemp protein–casein [21], the specific gelation behavior of casein with SPI remains underexplored. A systematic investigation into how the blending ratio and the inherent structural features of SPI dictate the functional properties and molecular mechanisms of these MTG-induced gels is needed, particularly because SPI’s distinct subunit composition suggests a gelation mechanism fundamentally different from those previously reported. To address this, we conducted a mechanistic investigation across a full range of casein-to-SPI mixing ratios, aiming to elucidate the specific intermolecular interactions and structural rearrangements that set this system apart from previously studied animal–plant protein blends.

Therefore, we hypothesize that casein will enhance SPI gel networks primarily through MTG-catalyzed covalent cross-linking, leading to improved mechanical properties and water retention in a ratio-dependent manner. The open structure of casein is expected to facilitate rapid and extensive cross-linking, potentially compensating for the less accessible reactive sites in SPI and resulting in a synergistic improvement in gel quality. This study aims to (1) formulate MTG-induced hybrid gels from casein and SPI at various blending ratios; (2) systematically characterize their microstructural, rheological, textural, and water-holding properties; and (3) elucidate the primary intermolecular interactions governing network formation and stability. The findings will provide fundamental insights into the design of novel, sustainable hybrid protein gels with tailored functionalities, thereby contributing to the development of next-generation hybrid food products.

## 2. Results and Discussion

### 2.1. Formation of Casein–SPI Hybrid Gels Induced by MTG

**Figure 1:**
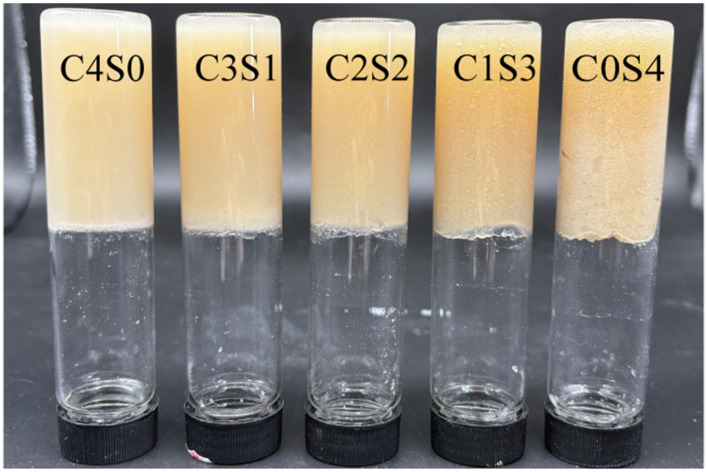
Appearance of casein and SPI gels. The resulting samples, coded as C4S0, C3S1, C2S2, C1S3, and C0S4, corresponded to casein–SPI mixing ratios of 4:0, 3:1, 2:2, 1:3, and 0:4 (*w*/*w*), respectively.

As shown in Figure 1, casein and SPI, when used individually, were capable of forming stable gels in the presence of MTG. These findings are consistent with previous reports demonstrating that MTG can induce gelation in plant and animal proteins by catalyzing ε-(γ-glutamyl)lysine isopeptide bond formation through the γ-carboxamide groups of glutamine residues, thereby promoting intra- and intermolecular cross-linking [22]. Moreover, the MTG-mediated reaction occurs under relatively mild conditions, whereas conventional thermal induction of protein gelation typically requires heating at 95 °C [23]. Even acid-induced gelation often involves a heat treatment step at 90 °C [24]. Notably, casein exhibits high thermal stability and typically requires heating above 100 °C to induce gelation [11,25]. Furthermore, replacing casein with SPI at varying ratios also resulted in the formation of homogeneous gels, suggesting that MTG can effectively induce covalent cross-linking between proteins from different sources.

### 2.2. Least Gelling Concentration

As shown in Table 1, for casein, a concentration of 8% was required to form a gel in the presence of MTG. In practice, the least gelling concentration of casein is primarily determined by the gel-induction method. For instance, when a combination of heat treatment and glucono-δ-lactone (GDL) was applied, casein formed a gel at a concentration of 10% [26]. In contrast, when MTG was used in conjunction with glucono-δ-lactone, gelation occurred at a casein concentration of 5% [27]. These results suggest that replacing heat treatment with MTG, in the presence of GDL, reduces the minimum gelling concentration of casein by half, underscoring the critical role of MTG in inducing casein gelation. This finding supports the use of MTG as the gelation inducer in this study. As shown in Table 1, SPI required a concentration of 8% to form a gel. In the mixed protein systems, gels were also formed at a total protein concentration of 8%, which matches the least gelling concentration (LGC) of both pure casein (C4S0) and pure SPI (C0S4) under the same MTG-induced conditions. This indicates that blending casein with SPI did not increase the overall LGC, suggesting that the gelation ability of the protein mixture was not impaired by the blending process. It is important to note that this 8% value represents the minimum concentration required for gel formation (the least gelling concentration). For all subsequent analyses aimed at evaluating the comprehensive properties of the gels (Section 2.3, Section 2.4, Section 2.5, Section 2.6, Section 2.7, Section 2.8, Section 2.9, Section 2.10, Section 2.11, Section 2.12 and Section 2.13), a total protein concentration of 10% was intentionally chosen. This concentration ensures the formation of gels with sufficient structural stability and mechanical integrity, thereby enabling reliable and consistent characterization across different formulations. A comparison with the literature further contextualizes this finding. In an MTG-induced lentil protein–casein system, a total protein concentration of 12% was required for gelation [20]. Under similar enzymatic conditions, our casein–SPI mixtures gelled at just 8%. Furthermore, unlike the hemp protein–casein system, which required the addition of glucono-δ-lactone alongside MTG to induce gelation [21], our casein–SPI system formed self-supporting gels with MTG alone. This notably lower LGC, achieved without the need for an additional gelling agent, suggests that SPI possesses a greater inherent susceptibility to MTG-mediated co-gelation with casein than either lentil or hemp protein. This difference underscores the profound influence of the plant protein source on network formation thresholds, highlighting the necessity for protein-specific investigations rather than generalizing findings across different plant-derived materials.

### 2.3. Free Amino Content

As shown in Table 2, MTG significantly reduced the free amino group content in both single-protein and mixed-protein systems, indicating that gel formation induced by MTG occurs through covalent cross-linking between proteins. This reduction confirms the involvement of ε-(γ-glutamyl)lysine isopeptide bonds. This finding is consistent with a previous study on hemp seed protein and casein, which also reported a decrease in free amino groups due to MTG-mediated covalent cross-linking between the two proteins [21].

### 2.4. Sodium Dodecyl Sulfate–Polyacrylamide Gel Electrophoresis (SDS-PAGE) Analysis

**Figure 2:**
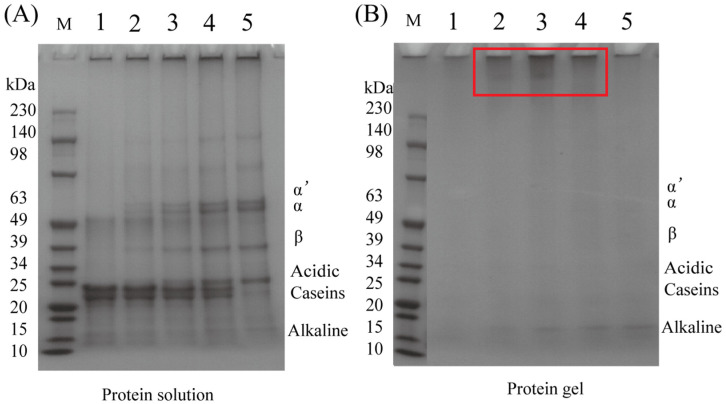
SDS-PAGE of casein–SPI mixtures (**A**) and gels (**B**). C4S0 (1), C3S1 (2), C2S2 (3), C1S3 (4), and C0S4 (5) presented that casein (C)-SPI (S) mixtures were prepared at mass ratios of 4:0, 3:1, 2:2, 1:3, and 0:4. M: protein markers. The red border indicates new polymer formations.

As depicted in Figure 2, compared to the protein solution, most subunits disappeared upon the addition of MTG, indicating the occurrence of intramolecular or intermolecular cross-linking, leading to the formation of high-molecular-weight polymers that were unable to penetrate the gel [21]. Notably, in the case of casein, all subunits became undetectable after MTG-induced gelation, implying that every subunit participated in cross-linking reactions. This observation is consistent with previous reports and explains the superior properties and microstructure of casein-based gels [28]. For SPI, alkaline subunits remained detectable in the gel samples, implying their exclusion from cross-linking. This discrepancy may arise from the location of alkaline subunits within the protein interior, which hinders their involvement in cross-linking formation during MTG-induced reactions [29]. This limited cross-linking of the alkaline subunit is also supported by earlier studies on SPI, which reported its continued presence in MTG-induced gels [30,31]. In contrast, acidic subunits, located on the protein surface, were more accessible and thus actively participated in cross-linking [32]. In mixed protein systems, the subunits involved in the cross-linking reaction were not observed, consistent with prior findings on hemp seed protein–casein [21] and lentil protein–casein gels [20]. However, intriguingly, new polymer formations were detected at the top of mixed gels (highlighted with red border), particularly evident in the C2S2 sample. This observation is consistent with the hypothesis that MTG catalyzes the formation of heteropolymers, likely representing covalent inter-protein cross-links between casein and SPI molecules, with the effect most pronounced at the 1:1 blending ratio. Mechanistically, this ratio may represent an optimal stoichiometric balance that maximizes both the spatial proximity and effective accessibility of reactive glutamine and lysine residues from each protein. These conditions favor MTG-mediated heterologous cross-linking, resulting in the most abundant formation of polymeric material. This finding further highlights the importance of investigating different protein ratios in hybrid gel systems.

### 2.5. Rheological Properties

**Figure 3:**
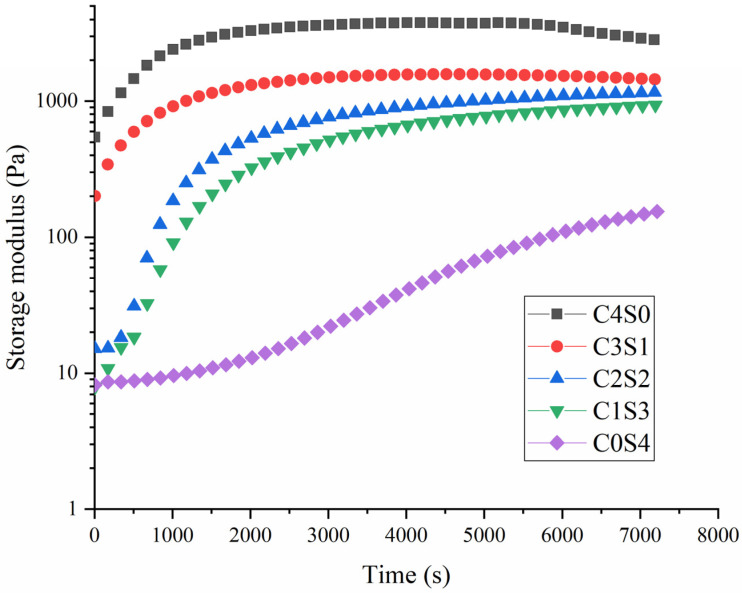
Time-sweep tests of casein and SPI gels. The resulting samples, coded as C4S0, C3S1, C2S2, C1S3, and C0S4, corresponded to casein–SPI mixing ratios of 4:0, 3:1, 2:2, 1:3, and 0:4 (*w*/*w*), respectively.

**Figure 4:**
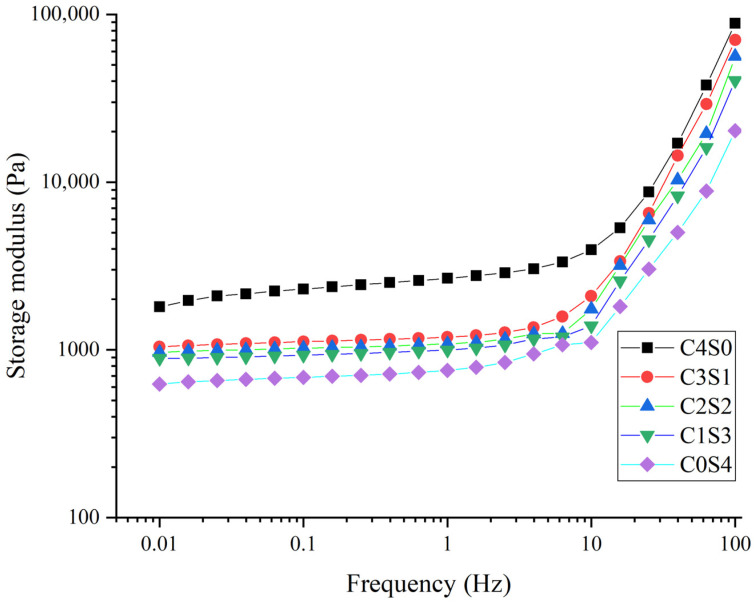
Frequency sweeps of casein and SPI gels. The resulting samples, coded as C4S0, C3S1, C2S2, C1S3, and C0S4, corresponded to casein–SPI mixing ratios of 4:0, 3:1, 2:2, 1:3, and 0:4 (*w*/*w*), respectively.

As shown in Figure 3, the storage modulus of all samples exhibited an increasing trend with prolonged incubation time, which further indicates that MTG can induce cross-linking reactions between protein molecules, thereby enhancing intermolecular interaction forces. It is noteworthy that casein displayed significantly higher storage modulus values compared to SPI during the incubation process. The casein system reached equilibrium at approximately 2000 s, whereas SPI required substantially longer incubation to achieve equilibrium. In contrast, the compact globular structure of SPI may hinder the accessibility of alkaline subunits to the enzyme’s active sites, consequently impairing gel network development. A previous study has similarly reported that the tightly folded conformation of plant globulins limits their susceptibility to MTG-mediated cross-linking [33]. For mixed protein systems, increasing the casein proportion not only accelerated the attainment of equilibrium storage modulus but also elevated the final modulus values, in agreement with prior findings on lentil protein–casein hybrid gels. Additionally, the frequency sweep analysis in Figure 4 corroborated that higher casein content corresponded to increased storage modulus, indicative of enhanced gel mechanical strength [34].

### 2.6. Textural Analysis

The textural properties of gels are critical for their practical applications, particularly in plant-based meat analogs and gel-based food products [35]. In this study, texture profile analysis (TPA) was employed to quantify two key mechanical parameters: hardness and springiness. Hardness, the peak force during the first compression, quantifies gel strength by measuring its resistance to deformation [36]. Springiness, or elasticity, gauges how well a gel returns to its original shape after force removal, a property closely tied to textural qualities like chewiness and mouthfeel [37]. As shown in Table 3, both hardness and springiness varied significantly depending on the casein-to-SPI ratio. Pure casein gels (C4S0) exhibited the highest hardness and springiness, while pure SPI gels (C0S4) showed the lowest values for both parameters, consistent with the rheological analysis presented in Section 2.5. Hybrid gels (C3S1, C2S2, and C1S3) displayed intermediate values, demonstrating that partial substitution of SPI with casein progressively enhanced the textural properties of the mixed systems. Notably, the gel prepared at a 1:1 casein-to-SPI ratio (C2S2) exhibited a significant increase in both hardness and springiness compared to the pure SPI gel, with improvements of nearly 79% and 66%, respectively. This marked enhancement indicates that the 1:1 ratio represents an optimal formulation in terms of both gel strength and elastic recovery. The improved hardness (gel strength) at this ratio can be attributed to casein’s greater susceptibility to MTG-catalyzed cross-linking. This cross-linking led to the development of a more compact and resilient protein network. The concurrent increase in springiness (elasticity) suggests that this network is not only stronger but also more flexible and resilient, capable of recovering its structure after deformation. These findings align with previous reports that strengthening intermolecular interactions is the key factor in improving gel texture [38].

### 2.7. Water-Holding Capacity

Water-holding capacity (WHC) refers to a material’s ability to retain water through capillary forces, with this capacity being largely determined by pore size [39]. Gels with superior water retention are generally more desirable for practical applications, as water loss can lead to structural collapse, shrinkage, hardening, and overall quality deterioration [40]. As shown in Table 3, the WHC of SPI gels was measured at 62.31%. With increasing casein content in the mixed protein system, the WHC of the hybrid gels progressively improved, with the sample prepared at a 1:1 ratio showing a nearly 35% improvement compared to the SPI gel, indicating that casein incorporation significantly enhances the water retention ability of SPI gels. The enhanced WHC likely results from covalent cross-linking between casein and SPI, which promotes the formation of a modified gel network with a more refined porous structure, thereby improving water immobilization through absorption and capillary forces [41]. Consequently, the microstructural characteristics of the gels will be investigated in a subsequent section. On the other hand, the WHC of SPI gels may also be influenced by intermolecular interactions. Typically, stronger intermolecular forces, including hydrogen bonds, ionic bonds, and hydrophobic interactions, enhance the gel’s ability to bind water molecules [42]. Therefore, the ability of casein to enhance SPI gel WHC likely stems from its promotion of these intermolecular interactions, which will be experimentally validated in subsequent studies.

### 2.8. Low-Field Nuclear Magnetic Resonance

**Figure 5:**
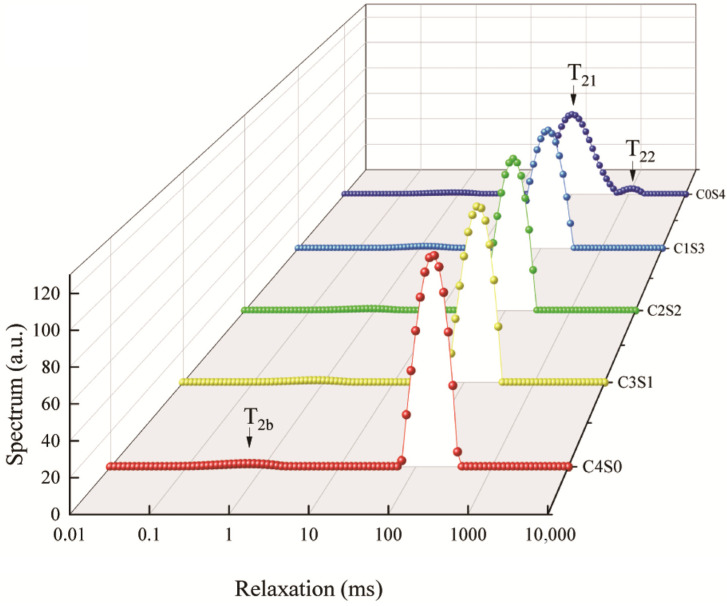
Low-field nuclear magnetic resonance of casein and SPI gels. The resulting samples, coded as C4S0, C3S1, C2S2, C1S3, and C0S4, corresponded to casein–SPI mixing ratios of 4:0, 3:1, 2:2, 1:3, and 0:4 (*w*/*w*), respectively.

Low-field nuclear magnetic resonance (LF-NMR) is an effective technique for monitoring water migration in materials, with the transverse relaxation time (T_2_) serving as a key parameter for characterizing different states of water. In T_2_ relaxation spectra, distinct peaks are generally assigned to three water populations: bound water (T_2b_, 0–10 ms), immobilized water (T_21_, 50–60 ms), and free water (T_22_, 1000–5000 ms) [43]. As shown in Figure 5, immobilized water constituted the dominant form across all gel samples examined. Notably, no free water was detected in casein gels, whereas a pronounced free water peak was observed in SPI gels. This finding is consistent with the lower WHC of SPI gels, as the presence of free water reflects weaker water retention. Notably, when casein was introduced as a partial substitute for SPI, the resulting hybrid gels showed no detectable free water, indicating that casein significantly enhances the water-binding capacity of the gel network. A previous study has also indicated that casein promotes stronger protein–water interactions, facilitating the immobilization of water molecules and contributing to a denser and more uniform gel structure, thereby improving WHC [44].

### 2.9. Secondary Structures

**Figure 6:**
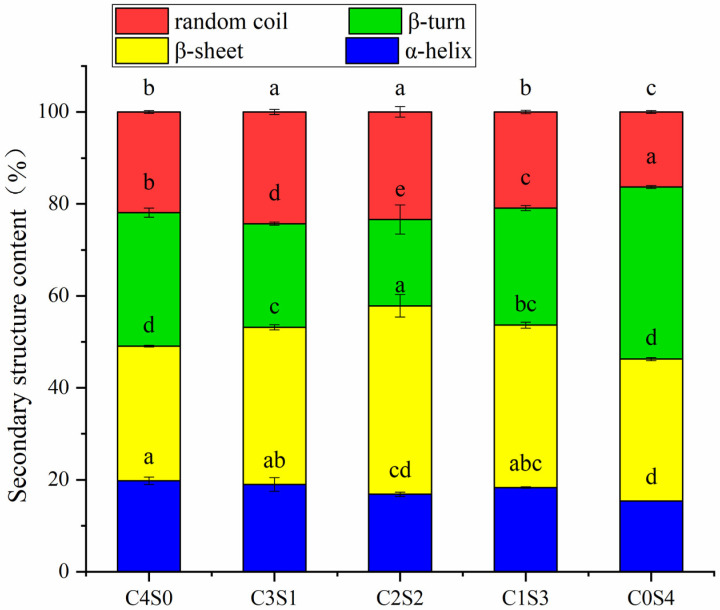
Secondary structures of casein and SPI gels. The resulting samples, coded as C4S0, C3S1, C2S2, C1S3, and C0S4, corresponded to casein–SPI mixing ratios of 4:0, 3:1, 2:2, 1:3, and 0:4 (*w*/*w*), respectively. Different letters in the same pattern represent significant differences (*p* < 0.05).

As shown in Figure 6, partial replacement of SPI with casein resulted in a significantly higher combined proportion of α-helix and β-sheet structures in the resulting mixed gels compared to the SPI gel. This suggests that the incorporation of casein enhances both the textural properties and WHC of SPI-based gels. The α-helices and β-sheets play important roles in stabilizing the gel microstructure, and their abundance is positively correlated with gel strength [26,45]. These ordered secondary structures enhance the rigidity and stability of polypeptide chains. Within the three-dimensional gel network, a higher proportion of such structured domains acts as reinforced, elastic junction points. This stabilizes the microstructure and promotes the formation of a more homogeneous matrix with a fine, interconnected pore structure. The resulting architecture strengthens capillary forces and increases available binding sites for water molecules, thereby restricting water mobility, reducing free water, and improving WHC. These results align with the concept that modifying the secondary structure of proteins is an effective strategy for improving gel properties [46]. Notably, at a casein-to-SPI ratio of 1:1, the hybrid gels showed a greater proportion of these ordered secondary structures compared to other ratios. This may be attributed to the formation of extensive casein–SPI conjugates catalyzed by MTG, as supported by SDS-PAGE analysis. Furthermore, this structural observation provides insight into why increasing the casein proportion beyond 1:1 did not lead to further significant improvements in gel strength, elasticity, or water-holding capacity.

### 2.10. Appearance and Microstructure

**Figure 7:**
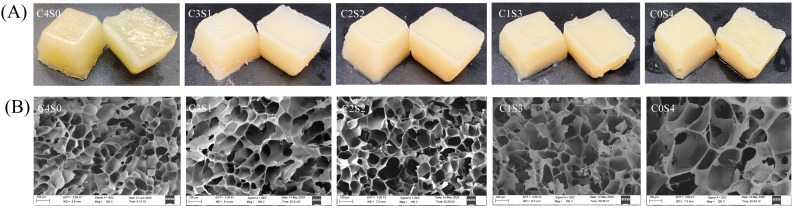
Appearance (**A**) and microstructure (**B**) of casein and SPI gels. The resulting samples, coded as C4S0, C3S1, C2S2, C1S3, and C0S4, corresponded to casein–SPI mixing ratios of 4:0, 3:1, 2:2, 1:3, and 0:4 (*w*/*w*), respectively.

As shown in Figure 7A, all gels exhibited self-supporting behavior, indicating that MTG successfully promoted the formation of structurally stable casein–SPI binary gels across all blending ratios. Furthermore, the gels showed a visible increase in stiffness with higher casein content. In the SPI gel samples, free water was observed at the bottom of the gel matrix. This phenomenon is consistent with the relatively low water-holding capacity and higher proportion of free water typically associated with SPI-based gels, as supported by the data presented in Table 3 and Figure 5. To further elucidate the microstructural characteristics of these gels, scanning electron microscopy (SEM) was performed on MTG-induced casein–SPI gels at various ratios (Figure 7B). The SPI gel exhibited a coarse structure with sheet-like aggregates. In contrast, as the proportion of casein increased, the gel network became more homogeneous and compact, with markedly smaller pores, suggesting an increase in cross-linking density. These structural observations align with trends observed in rheological, textural, and WHC measurements, indicating that the casein-to-SPI ratio plays an important role in defining gel architecture. The refined pore structure strengthens capillary forces within the matrix, thereby entrapping water and limiting its mobility. This interpretation is supported by the disappearance of the free water signal (T_22_) in the LF-NMR spectra (Figure 5). Partial incorporation of casein promoted the formation of a more ordered, fine-structured gel network, enhancing gel strength and demonstrating the potential of SPI as a functional component in hybrid and dairy-alternative food systems. These findings are consistent with a previous report on lentil protein and casein mixtures, in which casein was shown to improve the gelation behavior of plant proteins [20]. However, in lentil protein–casein mixed systems, key gel properties such as gel strength, springiness, and WHC were found to increase with rising casein content. This trend contrasts with our observations in the casein–SPI system, where the highest values for these properties were achieved at a 1:1 casein-to-SPI ratio; further increases in casein did not lead to significant improvements. This difference suggests that plant proteins from different sources exhibit distinct behaviors when forming hybrid gels with casein. The variation may arise from differences in protein composition and structure across plant sources, which influence the extent of cross-linking with casein and ultimately affect gel structure and functionality. Importantly, this finding has practical implications for balancing animal and plant proteins in formulations. The C2S2 (1:1) blend represents an optimized ratio that achieves high functionality while minimizing animal protein content. Rather than being a compromise, the 1:1 ratio emerges as a practically meaningful optimum—one where the structural advantages of casein are most efficiently leveraged to enhance SPI functionality. This aligns with the principle of using minimal animal protein for maximal functional improvement in sustainable food design.

### 2.11. Intermolecular Interactions

**Figure 8:**
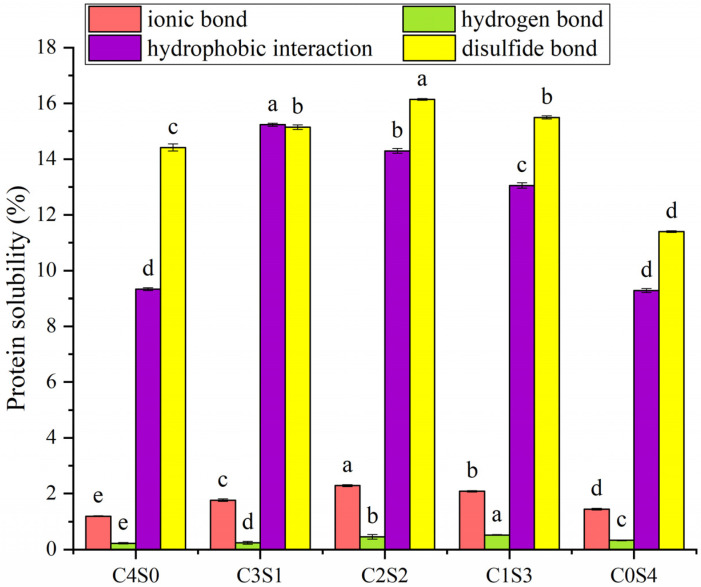
Intermolecular interactions of casein and SPI gels. The resulting samples, coded as C4S0, C3S1, C2S2, C1S3, and C0S4, corresponded to casein–SPI mixing ratios of 4:0, 3:1, 2:2, 1:3, and 0:4 (*w*/*w*), respectively. Different letters in the same pattern represent significant differences (*p* < 0.05).

The three-dimensional architecture of the gel is stabilized by a combination of intermolecular interactions, such as hydrophobic forces, hydrogen bonding, ionic associations, and covalent cross-links [47]. Gel solubility was examined in various solvent systems to better understand the forces stabilizing the three-dimensional gel network, primarily formed via MTG-catalyzed cross-linking. As illustrated in Figure 8, disulfide bonds and hydrophobic interactions were suggested to be major contributors to the network stability. This could be attributed to the fact that MTG induced intermolecular cross-linking between protein molecules, thereby further promoting their aggregation through hydrophobic interactions or facilitating the oxidation of free thiol groups to form disulfide bonds [48]. Compared to SPI gels, the incorporation of casein enhanced multiple intermolecular forces, including ionic bonds, hydrogen bonds, hydrophobic interactions, and disulfide bonds, which collectively contributed to the improved mechanical strength of the casein–SPI hybrid gels. This finding aligns with a previous study, which showed that the addition of bovine serum albumin to SPI promoted gel formation by enhancing intermolecular hydrophobic interactions and facilitating disulfide bond formation [49]. However, the enhancement in these forces did not exhibit a clear dose-dependent relationship with casein concentration, despite the continuous improvement in macroscopic gel properties. This suggests that gel stability in the composite system is not solely attributable to those interactions but is also critically influenced by covalent cross-links formed between SPI molecules, casein molecules, and casein–SPI complexes via MTG catalysis.

### 2.12. Mechanism of MTG-Induced Gel Formation in Casein–SPI Hybrid Systems

The enhanced gel properties at specific blending ratios (particularly 1:1 casein-to-SPI) appear to arise from a synergistic interaction between the two proteins. SDS-PAGE provides molecular evidence supporting that covalent cross-linking plays a central role in network formation. Casein’s open micellar structure exposes abundant glutamine and lysine residues, facilitating MTG accessibility. This is reflected in the near-complete disappearance of casein bands in all gel samples (Figure 2), indicating its high reactivity. In contrast, SPI’s compact globular structure sterically hinders access to many reactive sites. Consequently, the casein-to-SPI ratio governs the overall availability of MTG substrate sites. The 1:1 ratio likely strikes an optimal balance between casein’s inherent reactivity and spatial proximity to SPI, favoring heterologous cross-links between the two proteins, evidenced by the prominent high-molecular-weight polymers unique to the C2S2 sample (Figure 2). This cross-linking efficiency at the molecular level translates directly into improved macroscopic properties (such as gel strength, elasticity, and water-holding capacity) observed at the 1:1 ratio (Table 3). The substantial reduction in free amino groups (Table 2) quantitatively confirms extensive formation of ε-(γ-glutamyl)lysine isopeptide bonds across all formulations. Fourier transform infrared (FTIR) analysis further shows that casein incorporation, particularly at the 1:1 ratio, markedly increases the proportion of ordered α-helix and β-sheet structures (Figure 6). These ordered domains likely act as rigid junction zones within the three-dimensional network, reinforcing the gel structure and explaining the concurrent increases in storage modulus and gel firmness (Figure 3 and Figure 4). The molecular and secondary structural changes directly shape the gel microstructure. SEM imaging reveals that the 1:1 ratio yields a homogeneous, finely porous matrix (Figure 7B)—a morphology that enhances water retention by increasing capillary forces and available surface area for water binding. This interpretation is supported by LF-NMR data showing the disappearance of the free water (T_22_) signal in casein-containing gels (Figure 5), consistent with their higher WHC (Table 3). Taken together, these findings point to a hierarchical mechanism: MTG first catalyzes extensive covalent cross-linking between casein and SPI, establishing the initial network. This cross-linking promotes and stabilizes ordered secondary structures, which in turn guide the formation of a uniform, fine-pored microstructure during gelation. Hydrophobic interactions and disulfide bonds then act as additional stabilizing forces, helping to maintain a cohesive, water-retentive gel matrix.

### 2.13. Limitations and Translational Considerations

From an application perspective, the casein–SPI hybrid gels developed in this study show promise as functional ingredients for various sustainable food formats. Their enhanced gel strength and water-holding capacity make them suitable as texturizers or binding matrices in plant-based meat analogs, where improved juiciness and structural integrity are essential. Their fine, homogeneous network structure may also serve as a useful base for dairy alternatives (such as cheese-style products) requiring smooth consistency and high water retention. The use of MTG also enables gel formation under mild thermal conditions, helping preserve heat-sensitive nutrients and flavor compounds.

Translating these findings into commercial applications calls for closer attention to several practical parameters. For instance, it is worth asking whether the MTG dosage used here aligns with current industry practice and whether the enzyme level could be reduced through optimization of other processing conditions. Real food matrices, of course, are far more complex than the simplified model system studied. Lipids, polysaccharides, salts, or competing proteins may all interfere with MTG accessibility or alter gelation behavior in ways not captured here. There is also a formulation constraint worth noting: while casein clearly enhances SPI gelation, its use precludes application in strictly plant-based products.

When considering practical implementation, several limitations should be acknowledged to provide a balanced perspective. These include the additional cost associated with MTG, the potential allergenicity of casein for certain consumer groups, and the scalability of enzyme-induced gelation from laboratory to industrial production. Addressing these challenges will be important for future technology translation. It should also be noted that the stability of these hybrid gels under realistic food-processing conditions (such as variations in ionic strength, pH, and thermal exposure) was not examined in this study and warrants further investigation. Furthermore, while the synergistic improvement in gel properties strongly supports our proposed mechanism, the molecular-level evidence for heterologous cross-linking remains indirect. The techniques employed in this study (SDS-PAGE) cannot definitively distinguish between heteropolymers and homopolymers. Therefore, the formation of specific casein–SPI covalent bonds, though highly probable, remains a hypothesis requiring direct confirmation. Future research should therefore explore the kinetics and site specificity of MTG toward casein and SPI, evaluate the sensory and digestive properties of the gels, and systematically assess their behavior in complex food matrices. More direct analytical techniques, such as mass spectrometry or immunoblotting, are warranted to unequivocally confirm the formation and structural characterization of casein–SPI heteropolymers. Direct comparisons with traditional thermal or acid-induced gels are also needed to quantitatively benchmark the functional and energetic benefits of the MTG-based process. Finally, exploring additional animal–plant protein combinations may further advance the development of next-generation protein ingredients with tailored stability and functionality.

## 3. Conclusions

This study shows that MTG effectively catalyzes covalent cross-linking between casein and SPI, forming hybrid gels whose structural and functional properties are strongly dependent on the blending ratio. Incorporation of casein significantly enhanced gel strength, water-holding capacity, and microstructural homogeneity, while increasing the proportion of ordered secondary structures (α-helix and β-sheet). These improvements were most pronounced at the 1:1 casein-to-SPI ratio. Importantly, increasing the casein content beyond this 1:1 ratio yielded no significant additional improvements, underscoring that this specific ratio represents an optimal balance between the two proteins that maximizes cross-linking efficiency. Mechanistically, MTG-catalyzed covalent cross-linking established the primary gel network, which was further stabilized by disulfide bonds and hydrophobic interactions. Notably, the highest functional performance was achieved at a 1:1 casein-to-SPI ratio, indicating that an optimal balance between the two proteins maximizes cross-linking efficiency and network formation. Collectively, these findings confirm the central hypothesis that casein can compensate for the limited gelation potential of SPI through enzyme-induced heterologous cross-linking, offering a viable strategy for enhancing plant protein functionality while reducing reliance on animal-derived proteins in sustainable food systems.

## 4. Materials and Methods

### 4.1. Chemicals

Casein (refined grade, 92% purity, milk-derived), MTG (200 U/g activity), and o-phthalaldehyde (98%) were supplied by Shanghai Yuanye Biotechnology Co., Ltd. (Shanghai, China). SPI (90% purity) was provided by Shandong Yuwang Eco-Food Co., Ltd. (Yucheng, China). Marker (10–250 KDa) was purchased from Shanghai Epizyme Biomedical Technology Co., Ltd. (Shanghai, China). Loading buffers were sourced from Changzhou Boyi Biotechnology Co., Ltd. (Changzhou, China). β-Mercaptoethanol (99%) was obtained from Aladdin Biochemical Technology Co., Ltd. Glycerol (99%) was purchased from Shanghai Lingfeng Chemical Reagent Co., Ltd. (Shanghai, China). Coomassie Brilliant Blue G-250 (90%), sodium chloride (99%), urea (98%), potassium bromide (99%), and tris(hydroxymethyl)aminomethane (99%) were purchased from Sinopharm Chemical Reagent Co., Ltd. (Shanghai, China).

### 4.2. Preparation of Casein–SPI Hybrid Gels

Casein (C)-SPI (S) mixtures were prepared according to [20] at mass ratios of 4:0, 3:1, 2:2, 1:3, and 0:4 in distilled water. The mixture was stirred overnight at 4 °C, which yielded mixed protein solutions with a final concentration of 10% (*w*/*w*). These samples were designated as C4S0, C3S1, C2S2, C1S3, and C0S4, respectively. After adjusting the pH to 7.5 with 0.1 M HCl or NaOH under constant agitation, MTG (15 U/g protein) was added. The mixtures were then incubated at 50 °C for 2 h in a water bath to induce gelation. The resulting gels were promptly chilled in an ice bath to terminate the enzymatic reaction and stored at 4 °C for subsequent analyses. Each formulation was prepared in triplicate.

### 4.3. Determination of the Least Gelling Concentration

To determine the least gelling concentration (LGC) of casein–SPI mixtures, dispersions with total protein contents ranging from 2% to 16% (*w*/*w*) were prepared for each blending ratio (4:0, 3:1, 2:2, 1:3, and 0:4) [50]. After stirring overnight at ambient temperature, the pH was brought to 7.5 using 0.1 M NaOH or HCl. MTG (15 U/g protein) was then incorporated, and the mixtures were incubated at 50 °C for 2 h. Following incubation, the samples were immediately cooled on ice and kept at 4 °C for 24 h. Gel formation was confirmed by inverting the sample tubes; samples that did not flow were considered to have formed a gel. The LGC determination was performed in triplicate for each protein ratio.

### 4.4. Determination of Free Amino Group

Free amino groups were quantified by the o-phthalaldehyde (OPA) assay as described previously [34]. The OPA reagent was freshly prepared by dissolving 40 mg of OPA in 1 mL of methanol, then sequentially adding 2.5 mL of 20% (*w*/*v*) SDS, 25 mL of 0.1 M boric acid, and 100 μL of β-mercaptoethanol, and finally diluting to 50 mL with distilled water. For analysis, 4 mL of this reagent was mixed with 200 μL of sample solution (2 mg/mL) and incubated for 2 min at room temperature. Absorbance at 340 nm was recorded with a Spark 10M microplate reader (Tecan, Männedorf, Switzerland). A blank (water instead of sample) was used for baseline correction. A standard curve was constructed using L-leucine (Y = 0.1144x + 0.0002). Measurements were conducted in triplicate for each sample.

### 4.5. SDS-PAGE Analysis

Protein patterns of casein–SPI mixtures and their corresponding gels were examined by SDS-PAGE under reducing conditions (with β-mercaptoethanol) following a previously reported protocol [34], with slight modifications. Samples were prepared by mixing protein solutions (5 mg/mL) with 4× reducing or non-reducing loading buffer at a 3:1 ratio. For gel specimens, approximately 10 mg of gel was extracted with the same buffer (0.125 M Tris, 4% SDS, 20% glycerol, pH 6.85) overnight at room temperature to reach a final concentration of 10 mg/mL. All samples were heated at 100 °C for 10 min prior to loading. Electrophoresis was carried out on 4–20% precast polyacrylamide gels using a DYCZ-24DH system (Beijing Liuyi, Beijing, China) at a constant voltage of 140 V until the tracking dye reached the gel bottom. Gels were stained with 0.1% Coomassie Brilliant Blue R-250 for 2 h and destained with a methanol–acetic acid solution until bands became distinct. Images were captured with a gel documentation system.

### 4.6. Rheological Behavior Analysis

Rheological properties were assessed with a DHR-1 rheometer (TA Instruments, New Castle, DE, USA) fitted with a 25 mm parallel-plate geometry (gap 1000 μm). The viscoelastic properties of casein–SPI mixtures at various mass ratios (4:0, 3:1, 2:2, 1:3, and 0:4) were evaluated. To prevent moisture loss, samples were sealed with paraffin oil prior to measurement. The protein solution (10% *w*/*w*) was supplemented with MTG at a dosage of 15 U per gram of protein, thoroughly mixed, and then 4 mL of the solution was loaded onto the rheometer stage. Gel formation was induced under the conditions described in Section 4.2. Time sweeps were conducted at 50 °C, 1 Hz, and 0.04% strain (within the linear viscoelastic region). Subsequently, frequency sweeps from 0.01 to 10 Hz were carried out on the fully formed gels at 25 °C and 0.04% strain. All rheological measurements were performed in triplicate.

### 4.7. Texture Analysis

Textural parameters (hardness and springiness) were determined with a TMS-PRO texture analyzer (Food Technology Corporation, Sterling, VA, USA) as detailed in a previous study [51]. A cylindrical probe (12.7 mm diameter) compressed gel specimens (20 mm height) to 40% of their original height at a constant speed of 1.0 mm/s, with a trigger force of 0.5 N. Five replicates were tested for each gel formulation.

### 4.8. Measurement of Water-Holding Capacity (WHC)

Water-holding capacity was evaluated by centrifuging about 1 g of gel (weighed as m_1_) at 4000 rpm and 4 °C for 15 min [52]. The supernatant was decanted, and any remaining surface moisture was gently blotted with filter paper; the pellet was reweighed (m_2_). WHC was calculated as (m_2_/m_1_) × 100%. WHC measurements were carried out in triplicate for each sample.

### 4.9. Low-Frequency Nuclear Magnetic Resonance (LF-NMR) Analysis

Water mobility in the gels was probed by low-field nuclear magnetic resonance using a Niumag NM42-40H-I analyzer (Suzhou, China) operating at 42 MHz and 32 °C. Gel samples (1.5 g) were placed in 15 mm NMR tubes. Transverse relaxation (T_2_) was measured with the Carr–Purcell–Meiboom–Gill sequence under the following conditions: repetition time 4000 ms, echo time 0.4 ms, 2 scans, and 7000 echoes. Each gel sample was analyzed in triplicate using LF-NMR.

### 4.10. Fourier Transform Infrared (FTIR) Analysis

Infrared spectra of freeze-dried gels were recorded on a Nicolet 5700 FTIR spectrometer (Thermo Fisher Scientific, Waltham, MA, USA). Samples were prepared by grinding 1 mg of gel powder with 30 mg of KBr and pressing into transparent discs. Spectra were collected over 4000–500 cm^−1^ at 4 cm^−1^ resolution. For each formulation, three independent preparations were measured. The amide I region (1600–1700 cm^−1^) was analyzed with PeakFit v4.12 software: after baseline correction, second-derivative and Gaussian curve fitting were applied to resolve overlapping peaks. Secondary structure contents were estimated from the relative areas of the fitted components, with assignments based on the literature [53].

### 4.11. Microstructure Analysis

For microstructural observation, gel blocks (approximately 15 × 15 × 1 mm) were cut from the center, freeze-dried, and mounted on aluminum stubs. After gold sputter-coating, samples were imaged with a scanning electron microscope (Carl Zeiss AG, Oberkochen, Germany) at an accelerating voltage of 3 kV and a magnification of 100×.

### 4.12. Intermolecular Interactions Analysis

Intermolecular forces stabilizing the gel network were investigated by selective solubilization following a reported procedure with modifications [54]. Gel samples (0.5 g) were dispersed in 9 mL of four different solvents: (B1) 0.6 M NaCl; (B2) 0.6 M NaCl + 1.5 M urea; (B3) 0.6 M NaCl + 8 M urea; and (B4) 0.6 M NaCl + 8 M urea + 0.5 M β-mercaptoethanol. After shaking at room temperature for 12 h, the suspensions were centrifuged at 10,000× *g* for 30 min. Protein concentration in the supernatants was measured by the Coomassie Brilliant Blue method. The differences in protein solubility between B2 and B1, B3 and B2, and B4 and B3 were attributed to hydrogen bonds, hydrophobic interactions, and disulfide bonds, respectively, while solubility in B1 reflected ionic interactions. All solubility measurements were performed in triplicate.

### 4.13. Statistical Analysis

Data are expressed as mean ± standard deviation of at least three independent replicates (*n* specified in each section). Statistical comparisons were performed with SPSS 26.0 (IBM, USA) using one-way ANOVA followed by Tukey’s HSD post hoc test when significant differences were detected. A threshold of *p* < 0.05 was considered statistically significant. In figures and tables, distinct superscript letters denote significant differences among groups, and error bars represent SD.

## Figures and Tables

**Table 1:** The least gelling concentration of casein–soy protein isolate (SPI) mixtures induced by microbial transglutaminase.

Sample	Concentration (%)							
	2	4	6	8	10	12	14	16
C4S0	−	−	−	+	+	+	+	+
C3S1	−	−	−	+	+	+	+	/
C2S2	−	−	±	+	+	+	+	/
C1S3	−	−	±	+	+	+	/	/
C0S4	−	−	±	+	+	+	/	/

± semi-solid; + solid; − liquid; / concentration too high for complete dissolution. The resulting samples, coded as C4S0, C3S1, C2S2, C1S3, and C0S4, corresponded to casein–SPI mixing ratios of 4:0, 3:1, 2:2, 1:3, and 0:4 (*w*/*w*), respectively.

**Table 2:** Free amino content of casein–SPI mixtures and gels.

Content (mg/g)	Sample				
	C4S0	C3S1	C2S2	C1S3	C0S4
Protein solution	2.70 ± 0.12 ^a^	2.13 ± 0.11 ^b^	1.78 ± 0.04 ^c^	1.62 ± 0.05 ^d^	2.56 ± 0.04 ^e^
Protein gel	0.22 ± 0.01 ^cd^	0.27 ± 0.02 ^a^	0.27 ± 0.01 ^a^	0.24 ± 0.01 ^bc^	0.20 ± 0.01 ^d^

Different letters within the same row represent significant differences (*p* < 0.05). The resulting samples, coded as C4S0, C3S1, C2S2, C1S3, and C0S4, corresponded to casein–SPI mixing ratios of 4:0, 3:1, 2:2, 1:3, and 0:4 (*w*/*w*), respectively.

**Table 3:** Hardness, springiness, and water-holding capacity of casein and SPI gels.

Textural Properties	Sample				
	C4S0	C3S1	C2S2	C1S3	C0S4
Hardness (N)	4.27 ± 0.08 ^a^	3.61 ± 0.08 ^b^	3.54 ± 0.16 ^b^	2.37 ± 0.15 ^c^	1.98 ± 0.15 ^d^
Springiness (mm)	4.13 ± 0.13 ^a^	3.81 ± 0.16 ^b^	3.60 ± 0.19 ^b^	2.47 ± 0.48 ^c^	2.17 ± 0.04 ^c^
Water-holding capacity (%)	90.90 ± 0.70 ^a^	86.95 ± 3.22 ^b^	84.40 ± 1.26 ^b^	68.41 ± 0.39 ^c^	62.31 ± 3.18 ^d^

Different letters within the same row represent significant differences (*p* < 0.05). The resulting samples, coded as C4S0, C3S1, C2S2, C1S3, and C0S4, corresponded to casein–SPI mixing ratios of 4:0, 3:1, 2:2, 1:3, and 0:4 (*w*/*w*), respectively.

## Data Availability

The raw data supporting the conclusions of this article will be made available by the authors upon request.

## Abbreviations

The following abbreviations are used in this manuscript:

C	casein
SPI	soy protein isolate
MTG	transglutaminase
WHC	water-holding capacity
FTIR	Fourier transform infrared
SEM	scanning electron microscopy
LF-NMR	low-frequency nuclear magnetic resonance
LGC	least gelling concentration
C4S0	C-SPI mixtures were prepared at mass ratios of 4:0
C3S1	C-SPI mixtures were prepared at mass ratios of 3:1
C2S2	C-SPI mixtures were prepared at mass ratios of 2:2
C1S3	C-SPI mixtures were prepared at mass ratios of 1:3
C0S4	C-SPI mixtures were prepared at mass ratios of 0:4
